# Development of a Stability-Indicating RP-HPLC Method for the Determination of Rupatadine and its Degradation Products in Solid Oral Dosage Form

**DOI:** 10.3797/scipharm.1208-10

**Published:** 2012-10-01

**Authors:** Harshal Kanubhai Trivedi, Mukesh C. Patel

**Affiliations:** 1 Analytical Research Lab, Cadila Pharmaceutical Ltd, Dholka-387 810, Gujarat, India.; 2 P. S. Science and H.D. Patel Arts College, S.V. Campus, Kadi-382 715, Gujarat, India.

**Keywords:** Rupafin, Method validation, Forced degradation, Assay, Related substances, Chromatography, Impurities

## Abstract

A simple, sensitive, and reproducible reversed-phase high-performance liquid chromatography (RP-HPLC) method, coupled with a photodiode array detector, was developed for the determination of rupatadine (RUPA) and its related substances in pharmaceutical dosage forms. Chromatographic separation was achieved on the Hypersil BDS (150 x 4.6 mm, 5 μm) column with a mobile phase containing a gradient mixture of a buffer (acetate buffer pH-6.0) and solvent (methanol). The eluted compounds were monitored at 264 nm for the related substances and assay, the flow rate was 1.0 mL/min, and the column oven temperature was maintained at 50°C. The developed method separated RUPA from its four known and three unknown impurities within 15.0 min. Rupatadine was subjected to the stress conditions of oxidative, acid, base, hydrolytic, thermal, and photolytic degradation. Rupatadine was found to degrade significantly under oxidative stress conditions, and degrade slightly under acid, base, hydrolytic, thermal, and photolytic stress conditions. All impurities were well-resolved from each other and from the main peak, showing the stability-indicating power of the method. The developed method was validated as per the International Conference on Harmonization (ICH) guidelines. The developed and validated RP-HPLC method is LC-MS compatible and can be explored for the identification of eluted unknown impurities of RUPA.

## Introduction

Rupatadine fumarate (RUPAF) is 8-chloro-11-{1-[(5-methylpyridin-3-yl)methyl]piperidin-4-ylidene}-6,11-dihydro-5*H*-benzo[5,6]cyclohepta[1,2-*b*]pyridine (2*E*)-but-2-enedioate. RUPAF discovery, pre-clinical, and clinical development was performed by J. Uriach y Cia, S. A. [1], a Spanish pharmaceutical company. RUPA is a second generation, non-sedating, long-acting histamine antagonist with selective peripheral H_1_ receptor antagonist activity. It further blocks the receptors of the platelet-activating factor (PAF) according to *in vitro* and *in vivo* studies. Rupatadine possesses anti-allergic properties such as the inhibition of the degranulation_of mast cells induced by immunological and non-immunological stimuli, and the inhibition of the release of cytokines, particularly of the TNF in human mast cells and monocytes. It was launched in 2003 in Spain by J. Uriach y Cia, S. A., with the brand name of Rupafin. RUPAF has been approved for the treatment of allergic rhinitis and chronic urticaria in adults and children over 12 years old. The defined daily dose (DDD) is 10 mg orally. The efficacy of RUPA as a treatment for allergic rhinitis (AR) and chronic idiopathic urticaria (CIU) has been investigated in adults and adolescents (aged over 12 years) in several controlled studies, showing a rapid onset of action and a good safety profile even in prolonged treatment periods of a year.

Very few methods have appeared in the literature for the assay determination of RUPA in bulk and pharmaceutical dosage forms by high-performance liquid chromatography (HPLC) [2–4]. Pooja Ramanuj et *al.*[5] described the method validation of RUPA and Aspirine by using the HPTLC technique. Nogueira et *al.*[6] described the assay method validation of RUPA by using the Micellar Electrokinetic chromatography technique. Few spectrophotometric methods are available for the determination of the RUPA assay [7–9]. Few RP-HPLC methods are available for the determination of the RUPA *in vitro* study [10, 11]. Some titration methods are available for the assay determination of RUPA [12, 13]. Some LC-MS methods are available for the determination of RUPA in human plasma [14, 15]. The RP-HPLC method has been reported for the characterization of RUPAF impurities in the drug substance [16]. RUPA is not an official drug substance or drug product in the European Pharmacopoeia (Ph. Eur.) and United States Pharmacopoeia (USP). To the best of our knowledge, none of the currently available analytical methods can separate all of the known related compounds and degradation impurities in RUPA dosage forms. Furthermore, there is no stability-indicating HPLC/UPLC method reported in the literature for the determination of RUPA and its impurities in solid oral dosage form. It is, therefore, felt necessary to develop a new rapid, stability-indicating method for the determination of the assay and impurities in RUPA solid oral dosage form.

**Fig. 1.**
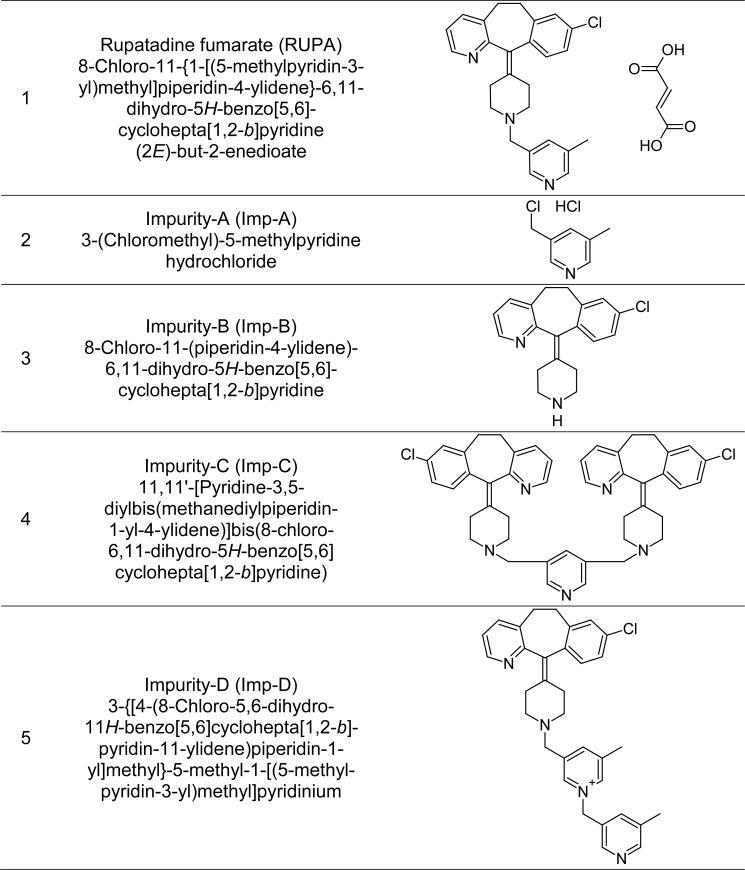
Chemical structures and IUPAC names of RUPA, Imp-A, Imp-B, Imp-C, and Imp-D.

Therefore, a reproducible stability-indicating RP-HPLC method was developed for the quantitative determination of RUPA and its degradation product (Imp-B), the separation of its other three known impurities (Imp-A, Imp-C, and Imp-D) and three unknown degradation products from each other and from RUPA within 15 min. This method is also useful for the mass determination of eluted unknown impurities. The developed method was successfully validated according to the ICH guidelines (Validation of Analytical Procedures: Test and Methodology Q2) [17]. In specificity study, Imp-B was found as a known degradation product and others were found as process impurities, so Imp-B was selected for the further validation parameters. The chemical structure and IUPAC name of RUPAF, Imp-A, Imp-B, Imp-C, and Imp-D are presented in Figure 1.

## Results and Discussion

### Method development and optimization

The main objectives of the RP-HPLC method development to rapidly assay and determine the related substances of Rupatadine in the pharmaceutical formulation were: the method should be able to determine the assay (AS) and related substances (RS) in a single sample preparation and should be accurate, reproducible, robust, stability-indicating, filter compatible, linear, free of interference from blank / placebo / impurities / degradation products, and straightforward enough for the routine use in a quality control laboratory.

The spiked solution of RUPA (1000 μg/mL), IMP-A (5 μg/mL), IMP-B (5 μg/mL), Imp-C (5 μg/mL), Imp-D (5 μg/mL), and fumaric acid was subjected to separation by RP-HPLC. Initially the separation of all compounds was studied using water as the mobile phase-A (MP-A) and acetonitrile (ACN) as the mobile phase-B (MP-B) on a HPLC column (Hypersil BDS, 150 x 4.6mm; 5.0μm) using a Waters system with the linear gradient program. The flow rate of 1.0 mL/min was selected with regards to the backpressure and analysis time as well. Various types of MP-A and MP-B were studied to optimize the method, which are summarized in Table 1 with the associated observations.

**Fig. 2.**
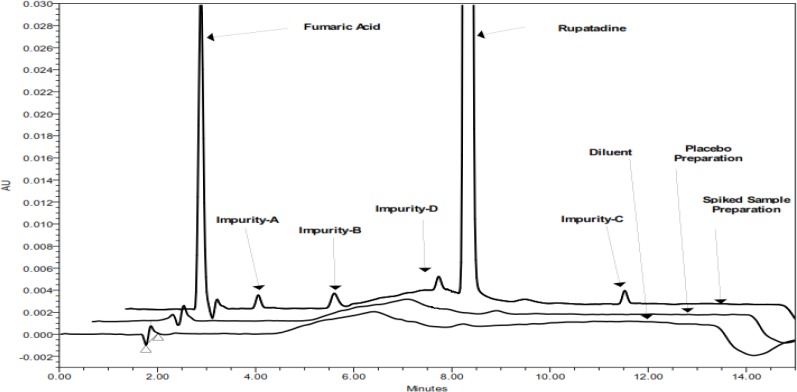
Overlaid chromatograms of blank, placebo, and spiked impurities along with sample

Based on the above mobile phase selection experimental study, the optimized HPLC parameters were; flow rate 1.0 mL/min; column oven temperature 50°C; ammonium acetate buffer (pH-6.0) as mobile phase-A, and methanol as mobile phase-B. In order to achieve symmetrical peak shape of all substances, more resolution between RUPA and Imp-D is needed. Finally, the desired separation (resolution not less than 2.0) with symmetrical peaks was obtained using the Hypersil BDS (150 x 4.6mm, 5.0μm) column. Column oven temperature was also studied and it was found that 50°C is more appropriate with respect to separation and peak shape. Based on the compounds’ UV response, 264nm (for the assay and related substances) was found to be more appropriate for the determination of RUPA and its impurities. RUPA, Imp-A, Imp-B, Imp-C, Imp-D, and fumaric acid were well resolved from each other and there was no chromatographic interference observed due to the blank and placebo in a reasonable time of 15.0 minutes [Figure 2].

### Analytical parameters and validation

After satisfactory development of the method, it was subjected to method validation as per the ICH guideline [17]. The method was validated to demonstrate that it is suitable for its intended purpose by the standard procedure to evaluate adequate validation characteristics (system suitability, accuracy, precision, linearity, robustness, solution stability, filter compatibility, and stability indicating capability).

### Specificity

Specificity is the ability of the method to measure the analyte response in the presence of its potential impurities. Forced degradation studies were performed to demonstrate the selectivity and stability-indicating capability of the proposed RP-HPLC method. Figure 2 shows that there is no interference at the RT (retention time) of RUPA due to the blank, placebo, and impurities. Stress studies were performed at a concentration of 1000 μg/mL of RUPA to provide the stability-indicating property and specificity of the proposed method.

Forced degradation studies were performed by the stress conditions, acid hydrolysis (0.1N HCl at 70°C for 24h), base hydrolysis (0.1N NaOH at 70°C for 24h), oxidation (5% H_2_O_2_ at 70°C for 4h), water hydrolysis (at 70°C for 24h), thermal (at 105°C for 48h), and photolytic (1.2 million lux hours/ 200 watt hours, square meter) to evaluate the ability of the proposed method to separate RUPA from its degradation products. Degradation was not observed when RUPA was subjected to acid, base, heat, photolytic, and hydrolytic conditions. Significant degradation was observed when the drug product was subjected to oxidative hydrolysis. The purity of the peaks obtained from the stressed sample was verified using the PDA detector. The obtained purity angle was less than the purity threshold for all of the stressed samples. An assay of samples was performed by comparison with reference standards, and the mass balance [% assay + % known impurities + area % unknown impurities] for each of the stressed samples was calculated. The results from the forced degradation study are given in Table 2.

### Precision

The system precision of the related substance method was verified by injecting six replicate injections of a standard solution containing RUPA (5 μg/mL). The % RSD (related standard deviation) of the peak area was calculated for RUPA (system precision). The method precision experiments were conducted in six individual preparations of the RUPA sample (1000 μg/mL) and the RSD (%) for the area percentage of Imp-B was calculated. Precision of the assay method was evaluated by performing six (n=6) independent assays of the RUPA tablet at the 100 μg/mL level against a qualified working standard. The RSD (%) of the six results was calculated. The intermediate precision of the assay and RS method was evaluated by different analysts, with different instruments, and on different days. The RSD (%) of the peak area of RUPA in system precision was within 1.0% (Table 3). The RSD (%) results of RUPA and its impurities for precision and intermediate precision are presented in (Table 4). These results confirmed the high precision of the method. As seen from this data, the acceptable system suitability parameters would be: resolution between Imp-B and RUPA is not less than 7.0, theoretical plates is not less than 8000, tailing factor for RUPA is not more than 2.0.

### Accuracy

The accuracy of an analytical procedure expresses the closeness of agreement between the true value and the observed value. The accuracy of the assay method for RUPA was evaluated in triplicate (n=3) at the three concentrations of 50, 100, and 150 μg/mL (50, 100 and 150%) of the drug product, and the recovery was calculated for each added (externally spiked) concentration. For impurity-B, the recovery was determined in triplicate (n=3) for 0.16, 2.0, 5.0, and 7.5 μg/mL (LOQ, 40, 100, and 150%) of the analyte concentration (1000 μg/mL) of the drug product, and the recovery of the impurities was calculated. The amount recovered was within ± 1.5 % (for the assay) and ± 5.0 % (for related substances) of the amount added, which indicates that there is no interference due to excipients present in the pharmaceutical dosage forms. It was confirmed from results that the method is highly accurate (Tables 5 and 6).

### Linearity of response

**Fig. 4.**
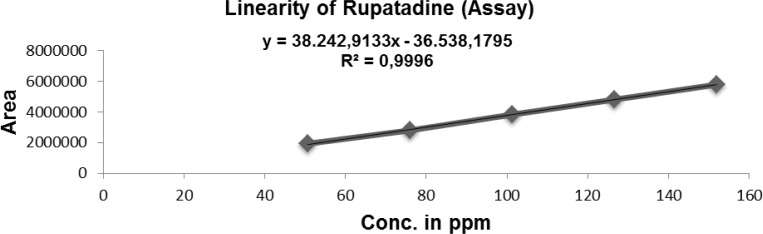
Linearity of Rupatadine (for Assay)

**Fig. 5.**
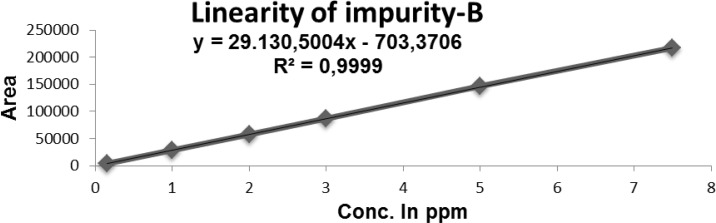
Linearity of Impurity-B

**Fig. 6.**
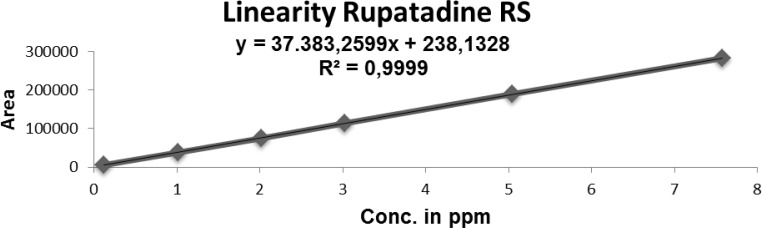
Linearity of rupatadine (for Related substances)

The linearity of an analytical method is its ability to elicit test results that are directly proportional, or by a well-defined mathematical transformation to the concentration of the analyte in a sample within a given range. The detector response linearity for Imp-B, and RUPA were assessed by injecting nine separately prepared solutions covering the range of LOQ (0.16 μg/mL) to 7.5 μg/mL (LOQ, 1.0, 2.0, 3.0, 5.0, and 7.5 μg/mL) of the normal analyte concentration (5 μg/mL). For the RUPA assay, the response function was determined by preparing standard solutions at seven different concentration levels ranging from 50 to 150 μg/mL (50, 75, 100, 125, and 150 μg/mL). The correlation coefficients, slopes, and *y*-intercepts of the calibration curve were determined (Table 7 and Figure 4). The correlation coefficient obtained was greater than 0.999 in both cases (Table 7 and Figures 4–6).

### Limit of detection (LOD) and limit of quantification (LOQ)

The LOD and LOQ for RUPA and its impurity were determined at a signal-to-noise ratio of 3:1 and 10:1, respectively, by injecting a series of dilute solutions with known concentrations. A precision study was also carried out at the LOQ level by injecting six (n=6) individual preparations and calculating the % RSD of the area for Imp-B and RUPA. The determined limit of detection, limit of quantification, precision at LOQ, and accuracy at the LOQ level for RUPA and Imp-B are presented in Table 8.

### Robustness

To determine the robustness of the method, the experimental conditions were deliberately changed. The resolution of RUPA and imp-B was evaluated. The effect of change in flow rate ± 0.1mL/min (0.9 and 1.1 mL/min), column oven temperature ± 5°C (45 and 55°C), mobile phase pH ± 0.2 units (5.8 and 6.2 pH), and wavelength ± 2.0 (262 nm and 266 nm) were studied. During the study, other chromatographic conditions were kept the same as per the experimental section. In all of the deliberately varied chromatographic conditions, all analytes were adequately resolved, and the order of elution remained unchanged. The robustness study obtained results which are presented in Table 9.

### Stability of solution

Drug stability in pharmaceutical formulations is a function of the storage conditions and chemical properties of the drug and its impurities. The conditions used in the stability experiments should reflect situations likely to be encountered during actual sample handling and analysis. Stability data is required to show that the concentration and purity of the analyte in the sample at the time of analysis corresponds to the concentration and purity of the analyte at the time of sampling. RUPA (1000 μg/mL)-spiked solution (with 5 μg/mL of Imp-B) was prepared in the diluent by leaving the test solutions at room temperature. The spiked solution was re-analyzed at 24h and 48h time intervals, and the assay and related substances were determinate for the compounds and compared against the fresh sample. The sample solution did not show any appreciable change in the assay and related substances value when stored at ambient temperature up to 48h; the data are presented in Table 10. The results from the solution stability experiments confirmed that the sample solution was stable for up to 48h during the assay and related substances determination.

## Experimental

### Materials and Reagents

The Rupatadine fumarate (100.9% w/w) working standard, Impurity-A (94.97% w/w), Impurity-B (99.74% w/w), Impurity-C (92.76% w/w), Impurity-D (87.22% w/w), placebo, and Rupatadine tablets were provided by Cadila Pharmaceutical Ltd., Ahmedabad, India. HPLC grade glacial acetic acid and methanol were obtained from J. T. Baker (NJ., USA). AR grade ammonium acetate and sodium hydroxide were obtained from Merck Ltd. (Mumbai, India). A 0.45 μm PVDF membrane filter and PVDF syringe filters were purchased from Pall Life Science Limited (India). 0.45 μm PVDF syringe filters were purchased from Millipore (India). High purity water was generated using the Milli-Q Plus water purification system (Millipore^®^, Milford, MA, USA). All other chemicals used were of analytical grade.

### Equipment

The Cintex digital water bath (Mumbai, India) was used for the specificity study. Photo stability studies were carried out in a photostability chamber (SUNTEST XLS+, ATLAS, Germany). Thermal stability studies were performed in a dry air oven (Cintex, Mumbai, India).

### Chromatographic conditions

Analyses were performed on the Waters HPLC^™^ system (Waters, Milford, USA), consisting of a binary solvent manager, sample manager, and PDA (photodiode array) detector. System control, data collection, and data processing were accomplished using Waters Empower^™^-2 chromatography data software. The chromatographic condition was optimized using the Hypersil BDS C8, (150 x 4.6 mm, 5 μm) column. The mobile phase-A consisted of an acetate buffer (adjusted pH to 6.0 with glacial acetic acid) and was filtered through a 0.45 μm nylon membrane filter. Methanol was used as the mobile phase-B. A mixture of methanol and water in a 50:50 ratio was used as a diluent. The final selected and optimized conditions were as follows: injection volume 20 μL, gradient (Table 11), flow rate of 1.0 mL/min, column oven temperature at 50°C, and detection wavelength at 264 nm for the assay and related substances determination. Under these conditions, the backpressure in the system was about 2,000 psi.

### System suitability solution preparation

The system suitability solution was prepared by dissolving the standard substance and Imp-B in diluent to obtain a solution containing 5 μg/mL of RUPA and 5 μg/mL of Imp-B.

### Standard solution preparation

The standard solution was prepared by dissolving the RUPA working standard in diluent to obtain a solution containing 100 μg/mL for the assay and 5μg/mL for related substances.

### Sample solution preparation

The sample solution was prepared by dissolving the sample (twenty tablets were crushed to a fine powder by mortar and pestle) in diluent to obtain a solution containing 1000 μg/mL of RUPA (for related substances) and 100 μg/mL (for the assay). It was then filtered through a 0.45 μm Nylon syringe filter and the filtrate was collected after discarding the first few milliliters.

## Conclusion

The rapid, gradient RP-HPLC method was developed for the quantitative and related substances analysis of Rupatadine in pharmaceutical formulation. Satisfactory results were obtained from the validation of the method. The run time (15 min) enabled rapid determination of RUPA. This method exhibited an excellent performance in terms of sensitivity and speed. This stability-indicating method can be applied for the routine analysis of production samples and to check the stability of Rupatadine in the bulk drug and formulation. Moreover, it can be applied for the determination of the assay, blend uniformity, content uniformity, and *in vitro* dissolutions of pharmaceutical products, where the sample load is higher and the high throughput is essential for faster delivery of results.

## Figures and Tables

**Fig. 3.**
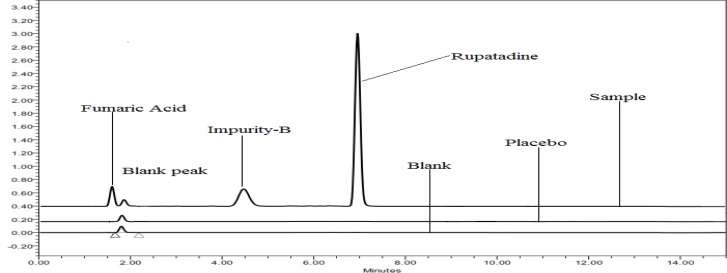
Overlaid specimens chromatograms of oxidative hydrolysis study

**Tab. 1.** Summary of mobile phase optimization

**MP-A**	**MP-B**	**Observation**
Water	CAN	Co-eluting peak of Imp-D and RUPA.
Water	MeOH	Co-eluting peak of Imp-D and RUPA.
Ammonium acetate buffer pH-6.0	MeOH	Good peak shape with better resolution

MeOH…Methanol.

**Tab. 2.** Summary of forced degradation results

**Degradation condition**	**Mass balance^#^**	**Purity of RUPA**		**Observation**
		**Angle**	**Threshold**	
Control sample	101.2%	0.312	0.362	NA
Acidic hydrolysis	97.8%	0.261	0.335	No significant degradation
Alkaline hydrolysis	99.0%	0.230	0.347	No significant degradation
Oxidation	102.0%	0.228	0.283	Significant degradation
Water hydrolysis	99.6%	0.298	0.355	No significant degradation
Thermal	99.9%	0.303	0.339	No significant degradation
Photolytic	98.3%	0.341	0.354	No significant degradation

NA… Not applicable;

#…% assay + % known impurities + area % unknown impurities

**Tab. 3.** System suitability results (system precision, method precision, and intermediate precision)

**Test**	**Parameters**	**RUPA (5 μg/mL)**	**Resolution (between RUPA and IMP-B at 5μg/mL)**	**RUPA (100 μg/mL)**
System precision	Area % RSD	0.2%	NA	0.2%
	USP resolution	NA	11.7	NA

Precision (n=6)	USP resolution	NA	13.1	NA
	Area % RSD	0.2%	NA	0.2%
	USP tailing	1.0	NA	1.8
	USP plate count	25365	NA	17040

Intermediate precision (n=6)	USP resolution	NA	13.1	NA
	Area % RSD	0.3%	NA	0.4%
	USP tailing	1.0	NA	1.8
	USP plate count	30274	NA	17050

**Tab. 4.** Precision (n=6) and Intermediate precision (n=6) results

**Substance**	**Precision**		**Intermediate precision**	

	**Mean %**	**% RSD**	**Mean %**	**% RSD**
RUPA (Assay)	101.2%	1.4%	99.4%	0.6%
Imp-B (RS)	0.50%	0.0%	0.51%	0.0%
RUPA Maximum unspecified impurity(RS)	0.07%	14.3%	0.05%	0.0%

**Tab. 5.** Accuracy results (Assay)

**Substance**	**At 50% (n=3)**		**At 100% (n=3)**		**At 150% (n=3)**	

	**%Recovery**	**%RSD**	**%Recovery**	**%RSD**	**%Recovery**	**%RSD**
RUPA (100μg/mL)	99.0%	0.1%	98.7%	0.3%	98.8%	0.4%

**Tab. 6.** Accuracy results (Related Substances)

**Substance**	**At 40% (n=3)**		**At 100% (n=3)**		**At 150% (n=3)**	

	**%Recovery**	**%RSD**	**%Recovery**	**%RSD**	**%Recovery**	**%RSD**
Imp-B (5μg/mL)	98.3%	0.3%	100.5%	0.3%	101.8%	0.3%
RUPA (5μg/mL)	100.8%	0.7%	100.2%	0.3%	100.0%	0.3%

Note: LOQ recovery reported with LOQ precision, refer table No 8

**Tab. 7.** Regression statistics

**Cpd.**	**Linearity range (μg/mL)**	**Correlation coefficient (r^2^)**	**Linearity (Equation)**	**Y-intercept bias at 100%**
RUPA	50 to 150	0.9996	y = 38,242.9x − 36,538.2	−1.0%
RUPA	0.12 to 7.5	0.9999	y = 37,383.3x + 238.1	0.1%
Imp-B	0.16 to 7.5	0.9999	y = 29,130.5x − 703.4	0.5%

**Tab. 8.** Results of LOD, LOQ, and LOQ precision (n=6)

	**RUPA**	**Imp-B**
LOD (μg/mL)	0.041	0.052
LOQ (μg/mL)	0.123	0.158
LOQ precision (% RSD)	2.0%	1.8%
LOQ Accuracy Mean (n=3)	0.7%	89.4%
LOQ Accuracy % RSD (n=3)	1.4%	100.8%

**Tab. 9.** Robustness study results

**Condition**	**Parameters**	**Between (Imp-B and RUPA)**	**RUPA** (5 μg/mL)	**RUPA** (100 μg/mL)
Normal methodology	USP resolution	13.1	NA	NA
	USP plate count	NA	30274	53592
	USP Tailing	NA	1.0	1.2
	% RSD	NA	0.3%	0.15
At flow rate 0.9 mL/min	USP resolution	12.4	NA	NA
	USP plate count	NA	31571	51503
	USP Tailing	NA	1.0	1.2
	% RSD	NA	0.3%	0.2%
At flow rate 1.1 mL/min	USP resolution	13.9	NA	NA
	USP plate count	NA	29062	17868
	USP Tailing	NA	1.0	1.5
	% RSD	NA	0.1%	0.1%
At 45°C column oven temp.	USP resolution	12.4	NA	NA
	USP plate count	NA	29865	15804
	USP Tailing	NA	1.0	1.4
	% RSD	NA	0.1%	0.1%
At 55°C column oven temp.	USP resolution	14.0	NA	NA
	USP plate count	NA	30966	17310
	USP Tailing	NA	1.0	1.4
	% RSD	NA	0.1%	0.1%
At mobile phase pH 5.8	USP resolution	12.2	NA	NA
	USP plate count	NA	25877	49841
	USP Tailing	NA	1.0	1.3
	% RSD	NA	0.3%	0.1%
At mobile phase pH 6.2	USP resolution	12.4	NA	NA
	USP plate count	NA	28633	51501
	USP Tailing	NA	1.0	1.2
	% RSD	NA	0.8%	0.2%
At wavelength 262nm	USP resolution	10.0	NA	NA
	USP plate count	NA	19449	17055
	USP Tailing	NA	1.1	1.5
	% RSD	NA	0.2%	0.2%
At wavelength 266nm	USP resolution	10.0	NA	NA
	USP plate count	NA	19454	17050
	USP Tailing	NA	1.1	1.5
	% RSD	NA	0.2%	0.4%

**Tab. 10.** Solution stability results

**Compound**	**0h**	**24h**	**48h**
RUPA (Assay)	99.9%	98.4%	98.9%
Imp-B	0.55%	0.55%	0.55%
Individual single unknown impurity	0.07%	0.07%	0.07%
Total impurities	0.70%	0.71%	0.71%

**Tab. 11.** Gradient elution program.

**Time in min**	**Mobile phase-A in %**	**Mobile phase-B in %**
0	40	60
2	40	60
4	20	80
11	20	80
12	40	60
15	40	60

## References

[1] Merlos M, Giral M, Balsa D, Ferrando R, Queralt M, Puigdemont A, García-Rafanell J, Forn J (1997). Rupatadine, a new potent, orally active dual antagonist of histamine and platelet-activating factor (PAF). J Pharmacol Exp Ther.

[2] Rupali LC, Mahajan MP, Swant SD (2012). Validation RP-HPLC method for the estimation of rupatadine fumarate in bulk and tablets dosage form. Der Pharma Chemica.

[3] Nareshkumar JM, Rakesh KJ, Rambir S, Keten GP RP-HPLC method development and its validation for assay of rupatadine fumarate in tablets dosage form. Pharma Analysis and Quality Assurance.

[4] Nogueira DR, D’Avila FB, Rolim CMB, Dalmora SL (2007). Development and validation of a stability-indicating LC method for the determination of rupatadine in pharmaceutical formulations. Chromatographia.

[5] Pooja R, Kashyap T, Hima S (2012). Analytical method development and validation for simultaneous estimation of rosuvastatin calcium and aspirin in bulk drug and capsule dosage form by HPTLC method. Pharma Analysis & Quality Assurance.

[6] Nogueira DR, da Silva SM, da Silva LM, Todeschini V, Dalmora SL (2008). Determination of rupatadine in pharmaceutical formulations by a validated stability-indicating MEKC method. J Sep Sci.

[7] Rele RV, Gurav PJ (2012). A Simple extractive spectrophotometric determination of Loratadine, Desloratadine and Rupatadine from pharmaceutical formulations. Int J Pharm Bio Sci.

[8] Rupali LC, Moreshwar PM, Sanjay DS (2012). Spectrophotometric estimation of rupatadine fumarate and montelukast sodium in bulk and tablet dosage form. Int J Pharm Pharm Sci.

[9] Patel PG, Vaghela VM, Rathi SG, Rajgor NB, Bhaskar VH (2009). Derivative spectrophotometry method for simultaneous estimation of rupatadine and montelukast in their combined dosage form. J Young Pharm.

[10] Dalmora SL, Nogueira DR, Calegari GZ, Bergamo AC, Stamm FP (2010). Development and validation of a dissolution test with reverced-phase liquid chromatography analysis for Rupatadine in tablets dosage forms. Quim Nova.

[11] Pushpendra AP, Margret C, Debjit B, Chiranjib BJ, Sampath KP (2009). Formulation and evaluation of fast dissolving tabalets of rupatadine fumarate. Der Pharmacia Lettre.

[12] Rele RV, Mahimkar SA, Sawant SA (2009). A validated simple titrimetric method for the quantitative determination of rupatadine as rupatadine fumarate from pharmaceutical dosages. Anal Chem Indian J.

[13] Goyal A, Sharma CS, Singh G (2010). Development of UV and Visible Spectrophotometric methods for estimation of rupatadine fumarate from tablet formulation. Int J of Pha Res and Dev.

[14] Wen J, Hong Z, Wu Y, Wei H, Fan G, Wu Y (2009). Simultaneous determination of rupatadine and its metabolite desloratadine in human plasma by a sensitive LC–MS/MS method. J Pharm Biomed Anal.

[15] Tian Y, Zhang J, Lin H, Liang J, Zhang Z, Chen Y (2008). High performance liquid chromatography-tandem mass spectrometric determination of rupatadine in human plasma and its pharmacokinetics. J Pharm Biomed Anal.

[16] Wang H, Ge P, Zhao W, Hang T, Li T (2009). RP-HPLC determination of rupatadine fumarate and its related substances. Chin J Pharm Anal.

[17] (2005). ICH, Validation of Analytical Procedure, Text and Methodology Q2(R1).

